# Cryogenic Focused Ion Beam Enables Atomic-Resolution Imaging of Local Structures in Highly Sensitive Bulk Crystals and Devices

**DOI:** 10.1021/jacs.1c12794

**Published:** 2022-02-14

**Authors:** Jinfei Zhou, Nini Wei, Daliang Zhang, Yujiao Wang, Jingwei Li, Xiaopeng Zheng, Jianjian Wang, Abdullah Y. Alsalloum, Lingmei Liu, Osman M. Bakr, Yu Han

**Affiliations:** † Multi-scale Porous Materials Center, Institute of Advanced Interdisciplinary Studies & School of Chemistry and Chemical Engineering, Chongqing University, Chongqing 400044, P. R. China; ‡ Physical Sciences and Engineering Division, KAUST Catalysis Center (KCC), King Abdullah University of Science and Technology (KAUST), Thuwal 23955-6900, Saudi Arabia; § Physical Sciences and Engineering Division, Advanced Membranes and Porous Materials (AMPM) Center, King Abdullah University of Science and Technology (KAUST), Thuwal 23955-6900, Saudi Arabia

## Abstract

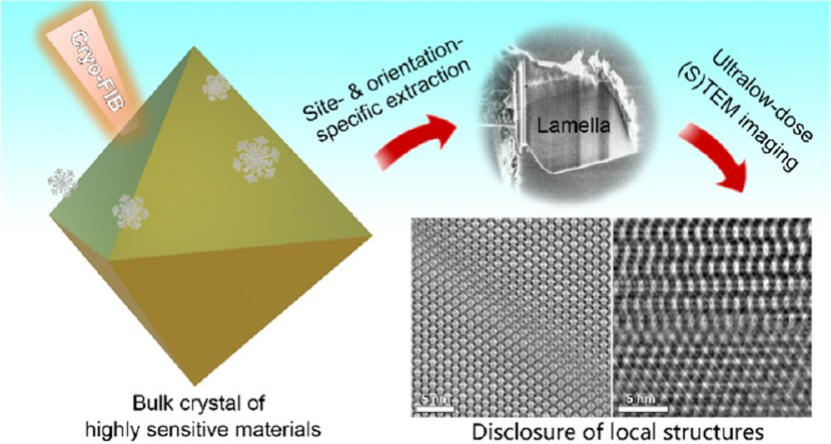

With the development of ultralow-dose (scanning) transmission electron microscopy ((S)TEM) techniques, atomic-resolution imaging of highly sensitive nanomaterials has recently become possible. However, applying these techniques to the study of sensitive bulk materials remains challenging due to the lack of suitable specimen preparation methods. We report that cryogenic focused ion beam (cryo-FIB) can provide a solution to this challenge. We successfully extracted thin specimens from metal–organic framework (MOF) crystals and a hybrid halide perovskite single-crystal film solar cell using cryo-FIB without damaging the inherent structures. The high quality of the specimens enabled the subsequent (S)TEM and electron diffraction studies to reveal complex unknown local structures at an atomic resolution. The obtained structural information allowed us to resolve planar defects in MOF HKUST-1, three-dimensionally reconstruct a concomitant phase in MOF UiO-66, and discover a new CH_3_NH_3_PbI_3_ structure and locate its distribution in a single-crystal film perovskite solar cell. This proof-of-concept study demonstrates that cryo-FIB has a unique ability to handle highly sensitive materials, which can substantially expand the range of applications for electron microscopy.

## Introduction

Transmission electron microscopy (TEM) is based on electron–matter interactions to probe the structural and chemical information of a specimen. The TEM specimens must be very thin to avoid multiple scattering of electrons.^1,2^ Although the acceptable thickness varies with the electron energy and atomic numbers of the sample, specimens thinner than 100 nm are generally required, and “thinner is better” is nearly an invariable axiom in TEM research. Therefore, specimen preparation is crucial for achieving high-resolution imaging and quantitative analysis when the sample is a bulk material. On the other hand, acquiring high-resolution TEM (HRTEM) images for electron-beam-sensitive materials, such as metal–organic frameworks (MOFs),^3−5^ supramolecular crystals,^6^ and organic–inorganic hybrid halide perovskites (HPs),^7,8^ is very challenging because their structures could easily be damaged by electron-beam irradiation. Under the low electron dose conditions required to preserve their inherent structures (typically <30 e^–^/Å^2^),^9^ conventional CCD cameras cannot produce usable images due to the poor signal-to-noise ratio. These beam-sensitive materials are traditionally considered unsuitable for TEM characterization; thus, no attention has been given to their specimen preparation.

Our recently developed ultralow-dose TEM technique, which combines a direct-detection electron-counting camera and suite of image acquisition and processing methods,^4^ has demonstrated the ability to obtain atomic-resolution images of various beam-sensitive materials without structural damage.^5,10−13^ Similar progress has been made in scanning TEM (STEM). For example, the emerging integrated differential phase-contrast STEM (iDPC-STEM) has proven effective for imaging beam-sensitive materials.^9,14−16^ However, nanosized crystals were directly imaged in these studies without any specimen thinning process.

Given that high-resolution imaging of electron-beam-sensitive materials has become feasible with the advances in ultralow-dose (S)TEM, it is time to explore how to prepare (S)TEM specimens of these materials to extend the application of new imaging techniques to bulk crystals. After all, in most cases, the materials we aim to analyze are in the form of bulk crystals rather than nanocrystals. However, electron-beam-sensitive materials are usually also sensitive to other forces. For instance, MOF crystals can be amorphized when strong mechanical force is applied; likewise, hybrid HPs may decompose when exposed to moisture or water.^17,18^ These materials are thus prone to structural damage during common specimen preparation processes, such as grinding, crushing, ion thinning, and ultramicrotomy (involving water).

Focused ion beam (FIB), which is a tool widely used in the semiconductor industry for fabrication, modification, and ablation of chips and devices, is also a standard method for preparing (S)TEM specimens.^19^ Compared with other methods, the unique advantage of FIB is that it allows site- and orientation-specific extraction of the specimen with nanometer-level precision. Although FIB is most often used to cut “hard” materials, FIB performed under cryogenic conditions (cryo-FIB) can be used to prepare specimens of “soft” materials or even those containing liquid, such as biological samples^20−22^ and electrode–electrolyte interfaces of lithium-metal batteries,^23−26^ in their native states. Moreover, cryo-FIB has also been used to prepare specimens for alloys,^27^ semiconductors,^28,29^ and inorganic thin-film solar cells.^30^ In these applications, cryo-FIB exhibited the advantages of reducing specimen contamination, inhibiting light element migration, and minimizing structural damage caused by ion beams.

In this work, we report that cryo-FIB can be used for preparing (S)TEM specimens of electron-beam-sensitive crystalline materials. We found that simply performing FIB at cryogenic temperatures can effectively lessen structural damage during ion-beam milling without the need for embedding the sample in vitrified ice. Using cryo-FIB, we prepared large-area high-quality specimens with the desired orientations from bulk MOF crystals and an HP-based solar cell device. These specimens enabled subsequent (S)TEM imaging to reveal three-dimensional (3D) local structures at an atomic resolution in these materials, which other methods could not discover.

## Results and Discussion

### Establishment of the Cryo-FIB Method for Highly Sensitive Materials

**Figure 1:**
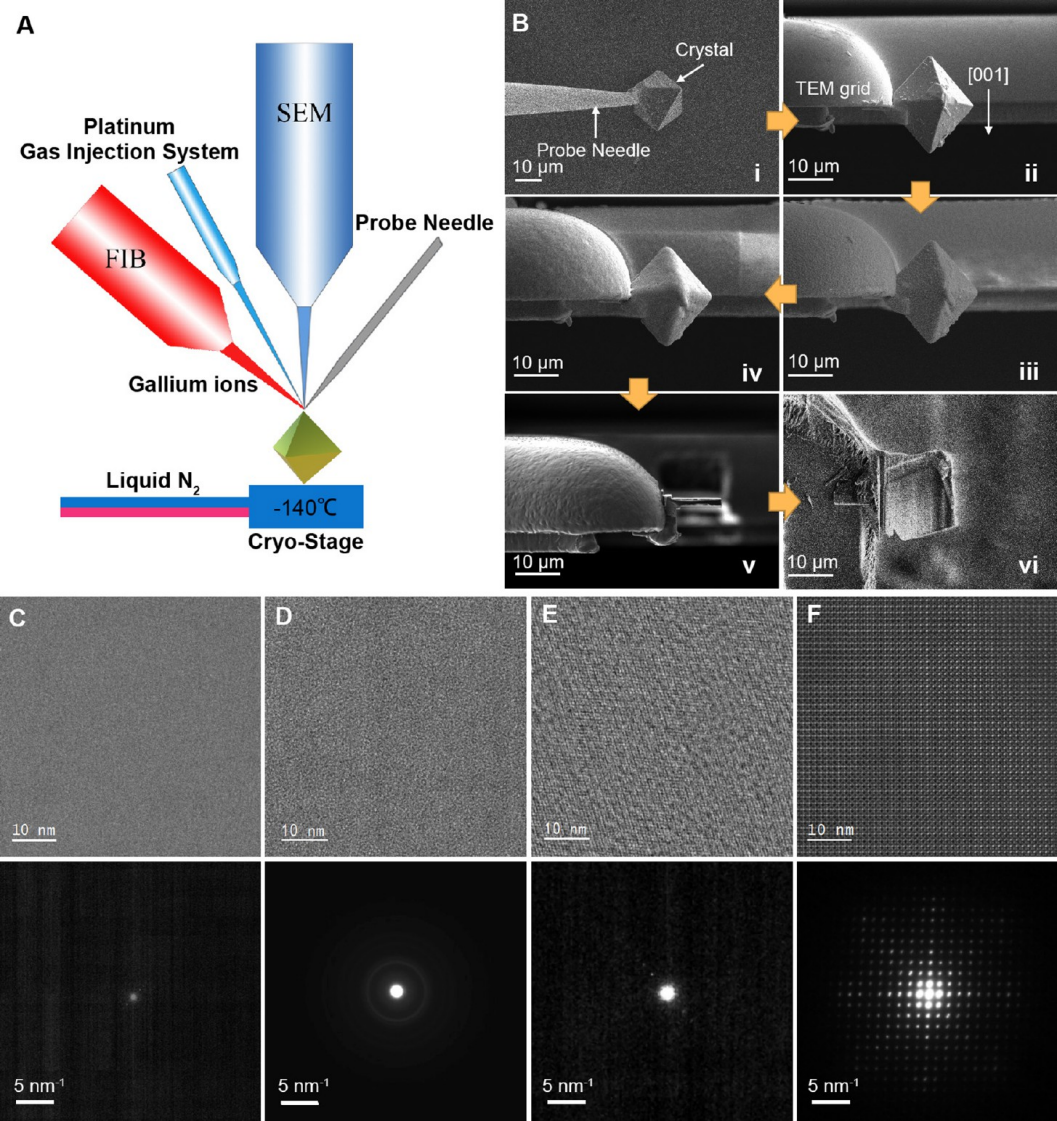
Cryo-FIB for preparing (S)TEM specimens of highly sensitive crystalline materials. (A) Schematic illustration of a cryo-FIB dual-beam system. (B) Typical cryo-FIB workflow illustrated using an HKUST-1 crystal of about 20 μm: (i) welding the crystal onto the probe needle, (ii) transferring the crystal to the TEM grid with the desired orientation, (iii) depositing the organometallic Pt precursor on the crystal surface, (iv) curing the Pt precursor using ion beams to form a Pt protection layer on the crystal surface, (v) coarse sectioning of the crystal into a lamella using ion beams, and (vi) fine ion-beam milling of the lamella into a thin slice (<100 nm), where the stage is tilted 52 degrees relative to (v). Steps (i) and (ii) are conducted at room temperature, and the remaining steps are conducted at −140 °C. (C–F) HRTEM images (upper) and SAED patterns (lower) of HKUST-1 specimens prepared at different conditions: (C) FIB operated at room temperature and (D–F) cryo-FIB using 5, 30, and 16 kV ion beams for fine milling, respectively.

To demonstrate the effectiveness and versatility of cryo-FIB, we conducted a series of case studies. We started with a very sensitive MOF (HKUST-1)^31^ to determine the optimal experimental conditions. The HKUST-1 consists of about 20 μm crystals that are too large to be directly imaged using (S)TEM. We tried to prepare a specimen of HKUST-1 by grinding but found it completely lost its crystallinity (see the Supporting Information, Figure S1). The cryo-FIB experiment was conducted on a dual-beam (focused ion beam and electron beam) system equipped with a cryo-stage that can be cooled down to −140 °C by passing liquid nitrogen (Figure 1A). The workflow included three main steps: (1) mounting the selected crystal on a TEM grid using a probe needle at room temperature (Figure 1B,i, and ii), (2) depositing and curing the organometallic Pt precursor to form a Pt-C protective layer on the crystal at −140 °C (Figure 1B,iii and iv), and (3) coarse sectioning and fine milling the crystal into a thin lamella using the ion beam at −140 °C (Figure 1B,v, and vi). The final specimen is a micron-scale wide and long slice, less than 100 nm thick, with the desired crystallographic orientation. The same procedure can be used for other sensitive materials with similar crystal sizes. The cryo-FIB procedure is slightly different for even larger samples (e.g., millimeter-sized crystals and devices), primarily in the first step. The detailed operational processes are described in the Supporting Information.

Some factors are critical to the success of the above-described cryo-FIB process. In the first step, the tilt angles of the probe needle, stage, and TEM grid must be precisely adjusted to obtain the desired orientation in the final specimen. In the second step, the cryogenic temperature significantly improves the adsorption efficiency of the gaseous precursor to quickly form a thick (∼5 μm) organometallic Pt layer within 1–2 min, ensuring that the MOF structure is fully protected during the subsequent ion-beam curing process. In contrast, conventional FIB operated at room temperature requires more than ten minutes to form the protective layer, even with the electron beam or ion beam to facilitate deposition. The long exposure to the beam irradiation can damage the MOF structure, as evidenced by the apparent crystal deformation (Figure S2). In the third step, the working temperature and accelerating voltage of the ion beam used for fine milling have important effects on the degree of structural damage. Regardless of the ion-beam conditions, operations at room temperature always destroy the crystallinity of HKUST-1, as revealed by the subsequent TEM and electron diffraction (ED) characterizations (Figure 1C). At −140 °C, three different accelerating voltages (30, 16, and 5 kV) were evaluated for fine milling with a fixed ion-beam current of 0.23 nA. The TEM/ED results revealed that the cryogenic temperature helped improve the stability of HKUST-1 during ion-beam milling, and the crystallinity was best maintained at 16 kV (Figure 1D–F).

**Figure 2:**
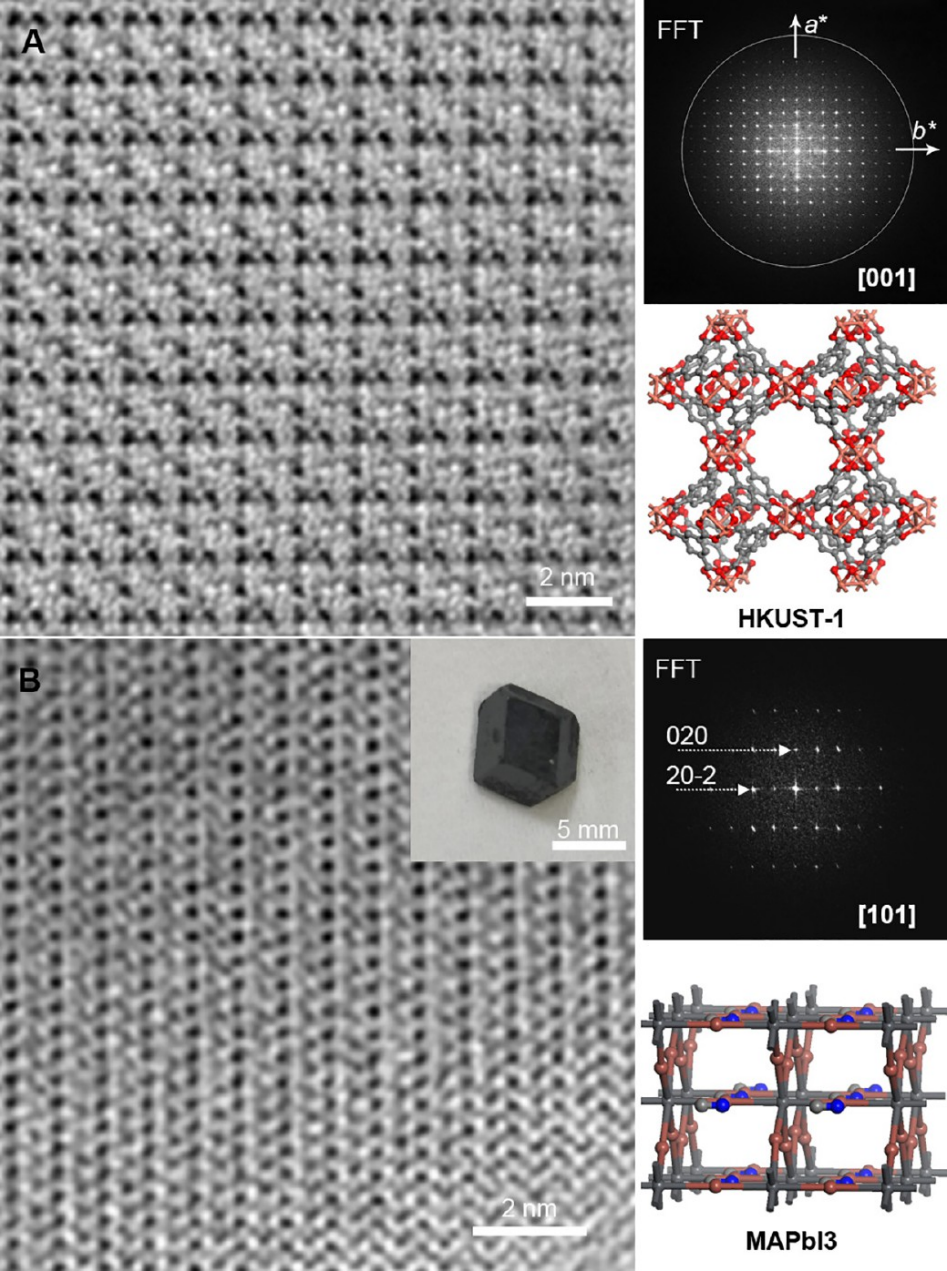
Ultralow-dose HRTEM of cryo-FIB-prepared specimens. (A) CTF-corrected HRTEM image of a specimen extracted from a 20 μm HKUST-1 crystal along the [001] zone axis and the corresponding FFT and structural model. The circle in the FFT represents an information transfer of 1.5 Å. (B) CTF-corrected HRTEM image of a specimen extracted from a millimeter-sized MAPbI_3_ crystal (see inset for photograph) along the [101] zone axis and the corresponding FFT and structural model.

An ultralow-dose HRTEM image was successfully acquired from the specimen prepared under the optimal conditions, with its Fourier transform exhibiting an information transfer of about 1.5 Å (Figure 1F). After correcting the effect of the contrast transfer function (CTF) of the objective lens, the image demonstrated good matching with the crystal structure of HKUST-1 projected along the [001] direction (Figure 2A). We applied the optimized cryo-FIB conditions to prepare a [101]-oriented specimen from a millimeter-sized HP CH_3_NH_3_PbI_3_ (MAPbI_3_) crystal and used ultralow-dose HRTEM to image the obtained specimen. The CTF-corrected image matched the structural model perfectly at a resolution of about 2.0 Å (Figure 2B). In contrast, conventional FIB operated at room temperature led to a crystal phase transition from MAPbI_3_ to PbI_2_ (Figure S3). These results demonstrate that the inherent crystal structures of highly sensitive materials, such as HKUST-1 and MAPbI_3_, can be well preserved during specimen preparation under appropriate cryo-FIB conditions.

### Structural Solution of the Planar Defects in MOF HKUST-1

Atomic force microscopy revealed that HKUST-1 crystals have an octahedral shape, exposing {111} facets and exhibiting surface fractures parallel to the crystal edges (i.e., the ⟨110⟩ directions).^32^ A subsequent confocal fluorescence microscopy study indicated that such fractures are formed by the {111} defect planes propagating from the crystal interior to the surface.^33^ However, as atomic force microscopy only provides surface information and confocal fluorescence microscopy has a low spatial resolution, the atomic structure of the defect plane is still unknown.

**Figure 3:**
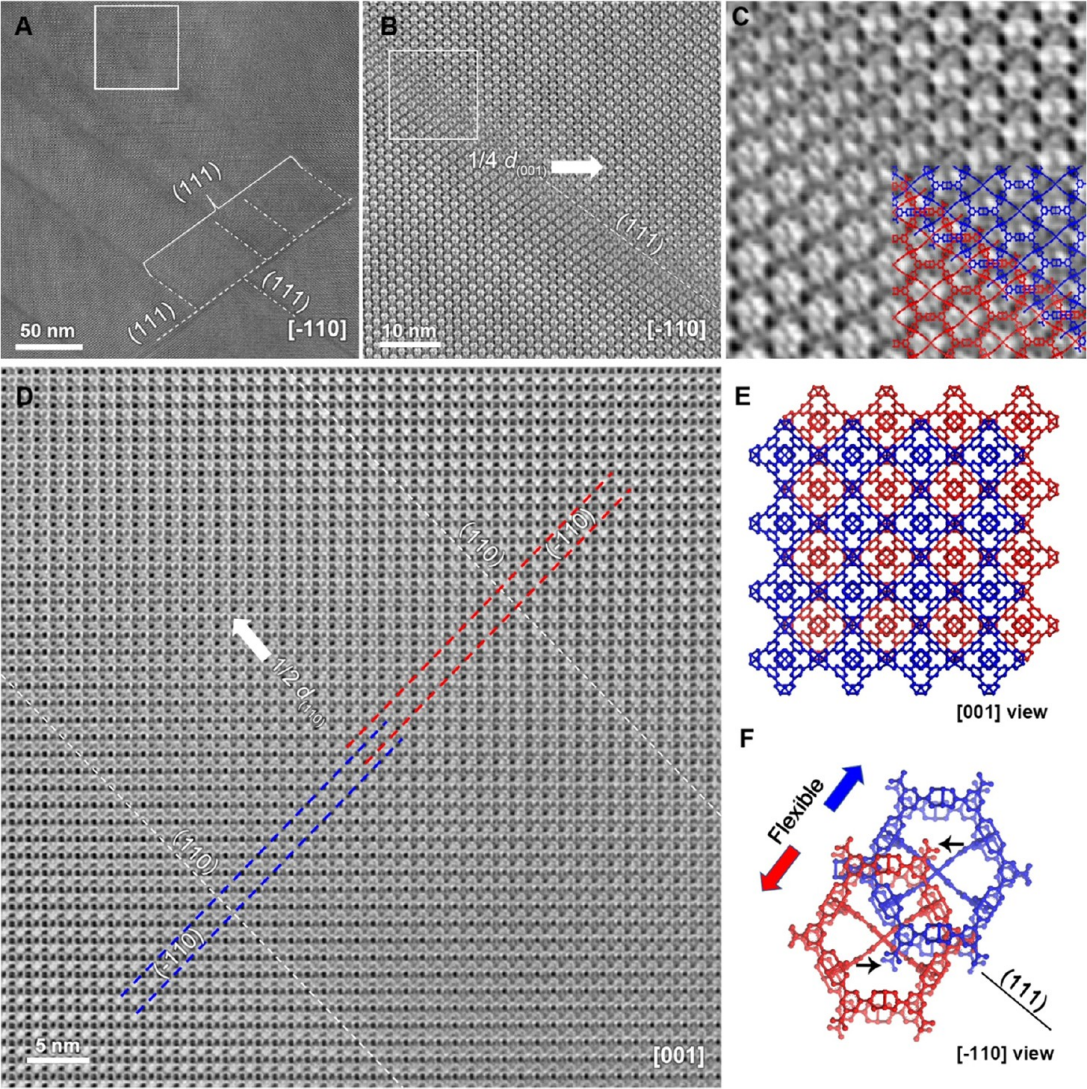
Planar defects in MOF HKUST-1. (A) CTF-corrected HRTEM image of a cryo-FIB-prepared specimen along the [1̅10] axis showing {111} defect planes. (B) Enlarged image from the marked area in (A) showing that the lattices at two sides of the defect plane are offset by 1/4 *a*. (C) Further enlarged image from the marked area in (B), superimposed with the proposed structural model. (D) CTF-corrected HRTEM image of cryo-FIB-prepared specimen along the [001] axis. In this direction, two sets of lattices (represented by blue and red lines) are offset by 1/4 (*a* + *b*), and their interpenetration spans tens of nanometers (the area between the two white dashed lines). (E, F) Structural model of the planar defect proposed based on HRTEM observations, where blue and red were used to differentiate the two sets of interpenetrating HKUST-1 structures. Black arrows in (F) indicate the resulting free carboxyl groups.

With the established cryo-FIB method, we can perform ultralow-dose HRTEM to observe the defective structure inside HKUST-1 crystals. Low-magnification TEM images taken from the ⟨110⟩-oriented specimen (Figures 3A and S4) display a high density of dark-contrast stripes, indicating abundant defects extending along the {111} planes. The CTF-corrected HRTEM image reveals that the lattices on both sides of the defect plane correspond to the perfect structure of HKUST-1 but are misaligned and offset by 1/4 *c* (Figure 3B,C). The defect area appears like an interface (approximately 1 × *d*_(111)_ thick) formed by the overlap of these two sets of lattices (Figure 3C). To understand the 3D structure of the defect, we carefully examined the ⟨100⟩-oriented and ⟨111⟩-oriented specimens. In the ⟨100⟩ direction, two sets of lattices are offset by 1/4 (*a* + *b*) and overlap each other in a region of tens of nanometers wide (Figure 3D). In the ⟨111⟩ direction, a perfect HKUST-1 structure was observed without defects (Figure S5). By combining the information from the three main zone axes, the formation of the defect plane can be understood as follows: along the {111} plane, a single crystal splits into two parts that are shifted relative to each other along the ⟨111⟩ direction by 1/4 (*a* + *b* + *c*), forming an interpenetrating structure at the interface (Figure 3E,F). For HKUST-1, this interpenetration leads to a distance of ∼3.2 Å between the aromatic rings of the 1,3,5-benzenetricarboxylate ligands at the interface, which is within the reasonable range of typical π–π stacking distances. We surmise that the interpenetration depth should be flexible, allowing this distance to be fine-tuned to a certain extent (Figure S6). Based on these results and analyses, we construct a structural model for planar defects frequently observed in HKUST-1 (see Supporting.pdb file 1).

Unlike common planar defects such as twin boundaries or stacking faults, the discovered interpenetrating boundaries lock adjacent grains through weak interactions, while creating disconnected interfaces. Consequently, there are abundant terminal carboxyl groups at the interpenetrating boundaries (Figure 3F), which accounts for the Brønsted acidity exhibited by HKUST-1 in catalysis.^33^

### Identification and 3D Reconstruction of a Concomitant Phase in MOF UiO-66

**Figure 4:**
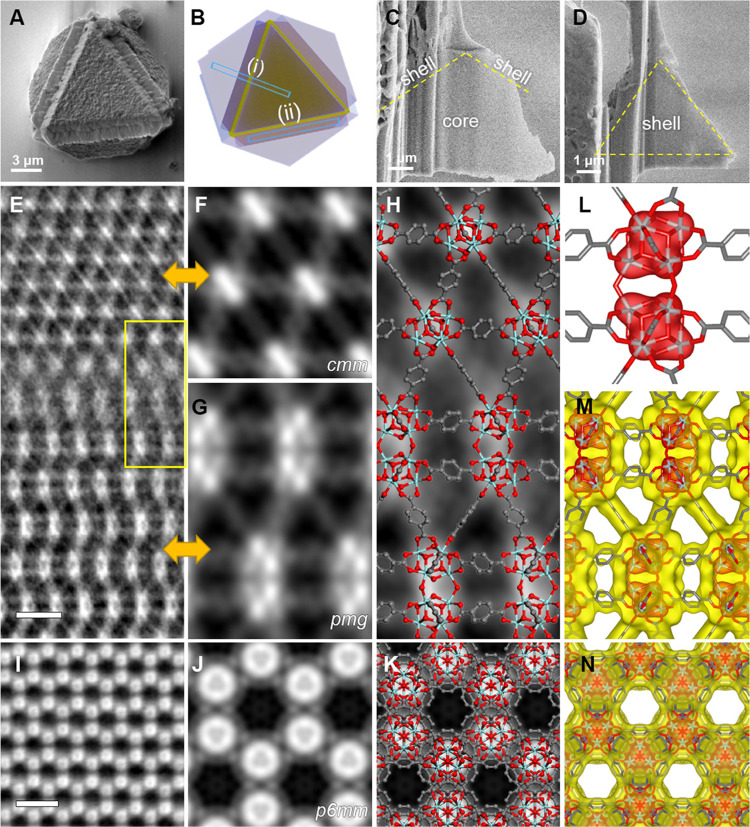
Intergrowth of two Zr-BDC MOFs. (A) SEM image of an octahedral MOF crystal with each triangular surface covered by a shell. (B) Schematic illustration of the core–shell structure. The two marked areas indicate where the specimens were extracted using cryo-FIB. (C, D) SEM images of the specimens from areas (i) and (ii) in (B), respectively. (E) iDPC-STEM image of the core–shell interface in (C). The raw image was cropped and rotated. (F, G) Symmetry-imposed lattice-averaged images of the core (along the [110] axis of cubic UiO-66) and shell (along the [100] axis of hexagonal Zr_12_-BDC) regions, respectively. (H) Enlarged image of the interfacial area marked by the yellow rectangle in (E), superimposed with a structural model. (I) iDPC-STEM image of the specimen in (D), along the [001] axis of hexagonal Zr_12_-BDC. (J) Symmetry-imposed lattice-averaged image of (I). (K) Enlarged image of (I), superimposed with the structural model. (L–N) Reconstructed 3D electrostatic potential map viewed in the [100] (L, M) and [001] (N) directions, with the structural model of Zr_12_-BDC superimposed. In (L), the high electrostatic potential corresponding to Zr atoms is clearly resolved, showing the precision of the reconstruction. The scale bars in (E) and (I) represent 2 nm.

Our attempt to synthesize large single crystals of MOF UiO-66(Zr) in a mixture solution of *N*,*N*-diethylformamide and formic acid^34^ unexpectedly resulted in the formation of crystals with abnormal morphology (Figure 4A). Unlike conventional UiO-66, which has a regular octahedral crystal shape, the obtained sample consists of core–shell-structured crystals, where the core is an octahedron (about 10 μm), and the shell (500–600 nm thick) grows on each triangular surface of the octahedron (Figures 4B and S7). Powder X-ray diffraction revealed several unidentifiable peaks in addition to those associated with UiO-66 (Figure S8A). Based on these observations, we speculated that the core and shell have different MOF structures.

To verify this speculation and determine the secondary phase and interfacial structure, we first used cryo-FIB to extract a specimen across the interface perpendicular to the ⟨110⟩ direction of the octahedron (Figure 4C). Ultralow-dose iDPC-STEM indicated that the two sides of the interface have distinctly different structures (Figure 4E). The core region has ordered triangular channels encompassed by three Zr clusters and three 1,4-benzenedicarboxylic acid (BDC) linkers, in good agreement with the ⟨110⟩-projected structure of UiO-66 (Figure 4F). The shell region also exhibits a porous structure but comprises different metal clusters double the size of the Zr_6_ clusters in UiO-66, and its plane symmetry group of the projection is different from that of UiO-66 (*pmg* vs *cmm*). From the interface outward, the characteristic ABAB stacking structure formed by the large clusters and their connections through BDC linkers is clearly identified (Figure 4G). To determine the 3D structure of this non-UiO-66 phase, we again employed cryo-FIB to extract another specimen from the shell region (not involving the core) parallel to the {111} surface of the octahedron (Figure 4D). The ultralow-dose iDPC-STEM image of this specimen indicates that large clusters are hexagonally arranged with a projection symmetry of *p*6*mm* (Figure 4I–K).

By combining the images from two projections (Figure 4G,J), the crystal structure of the shell is determined to be hexagonal with *a* = 13.5 Å and *c* = 37.2 Å, and the two images can be indexed to the [100] and [001] zone axes, respectively. Based on the observed reflection conditions (*hhl*: *l* = 2*n*; 00*l*: *l* = 2*n*) and plane symmetry groups ([001]: *p*6*mm*; [100]: *p*2*gm*), the space group is determined to be **P**6_3_/**mmc** (194). The electron crystallography technique was used to render a 3D electrostatic potential map of the structure from the Fourier summation of the crystal structure factors determined from the iDPC-STEM images (Table S1 and Figure S9). The reconstructed potential map presents an *hcp* structure comprising Zr_12_ clusters (dimers of the Zr_6_ clusters in UiO-66; Figure 4L). Each Zr_12_ cluster is connected to six clusters in the same layer (the *a*–*b* plane), three clusters in the upper layer, and three clusters in the lower layer, through a total of 18 BDC linkers (Figure 4M,N). The powder X-ray diffraction pattern of the core–shell crystals can be well indexed based on the coexistence of UiO-66 and the determined structure, and the refined unit cell parameters of the hexagonal phase are *a* = 14.17 Å and *c* = 36.2 Å (Figure S8B). The interfacial structure between the core and shell can also be derived based on the two bulk structures and the iDPC-STEM image contrast (Figure 4H and Supporting.pdb file 2).

Through the literature search, we found that the determined hexagonal structure has been reported as an independent MOF (denoted Zr_12_-BDC).^35−37^ Nevertheless, our research fully demonstrates that the combination of cryo-FIB and ultralow-dose imaging can determine the 3D structure of the concomitant minor phase in highly sensitive materials. This unique ability is based on the characteristics of cryo-FIB, which can extract specimens in desired locations and orientations without destroying the inherent structure. It is conceivable that, when using other crystal structure characterization techniques, it is almost impossible to accurately locate minor phases in micron-sized crystals or collect data from multiple specified orientations, especially for highly sensitive materials.

### Unraveling Structural Inhomogeneity in Single-Crystal Perovskite Solar Cells

The structural heterogeneity in hybrid halide perovskite solar cells has significant impacts on their performances, thus attracting extensive research interest.^38^ For instance, the nature of grain boundaries in polycrystalline perovskite thin films has been carefully investigated by various methods,^39−41^ including direct imaging using low-dose low-angle annular dark-field STEM.^42^ Single-crystal halide perovskites are generally considered to have no grain boundaries and a low density of defects to trap charge carriers.^43−46^ However, whether halide perovskite “single crystals” have local structural inhomogeneities has largely remained unexplored because of their extremely high sensitivity and large sizes.

**Figure 5:**
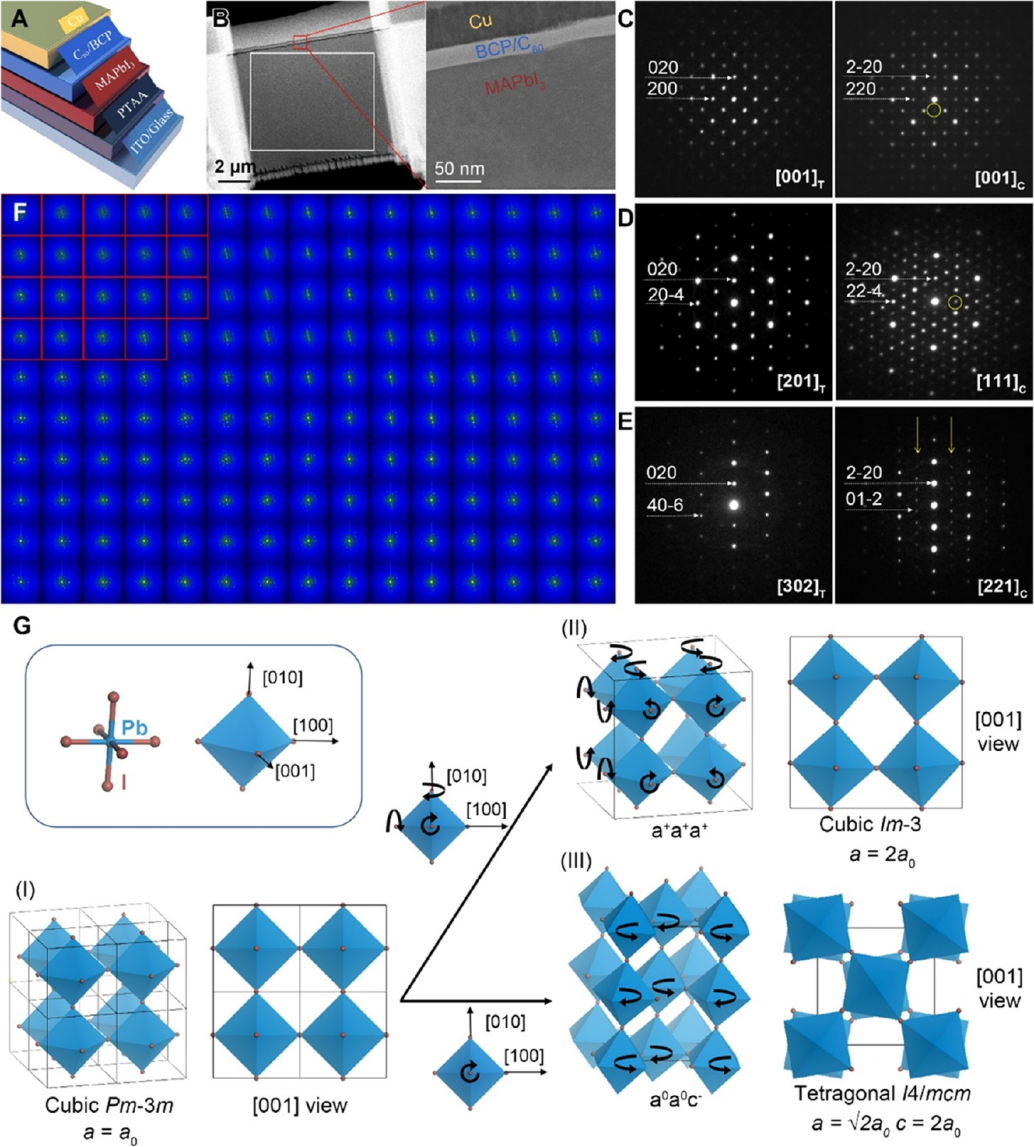
Electron diffraction study of a single-crystal MAPbI_3_ solar cell. (A) Schematic illustration of the planar configuration of the solar cell. (B) Low-magnification images of the cryo-FIB-prepared specimen, presenting the lamellar cross-sectional structure of the solar cell. (C–E) Representative SAED patterns from three cryo-FIB-prepared specimens. In each panel, the left pattern is indexed according to the primary tetragonal structure, whereas the right pattern containing super-reflections (indicated by yellow circles and arrows) is indexed according to the secondary cubic structure. (F) SAED mapping of the area marked by the white rectangle in (B), where the SAED patterns with super-reflections are labeled with red boxes. (G) Derivations of the cubic *Im*3̅ phase (II) and tetragonal *I*4/*mcm* phase (III) from the parent cubic *Pm*3̅*m* phase (I) by varying the tilting mode of PbI_3_ octahedrons.

To elucidate this issue, we combined cryo-FIB with ED to investigate a single-crystal MAPbI_3_ perovskite solar cell with a p–i–n planar configuration (Figure 5A). Specifically, cryo-FIB was employed to extract three cross-sectional specimens from the solar cell along different directions. One of these specimens is displayed in Figure 5B, involving the electrode (Cu) layer, buffer (BCP) layer, electron-transport (C60) layer, and a large area of the single-crystal MAPbI_3_ layer (approximately 8 × 6 μm). The subsequent ED studies were focused on the MAPbI_3_ layer.

Similar to classic inorganic perovskites, the organic–inorganic hybrid perovskite MAPbI_3_ has a series of structural variants. Their structures can be understood as derived from the parent cubic structure (*Pm*3*®m*, *a* = 6.35 Å) by tilting the PbI_3_ octahedrons around the [100], [010], and [001] axes in different ways (described by Glazer’s notation).^47^ The three most common structures of MAPbI_3_ are cubic (*Pm*3̅*m*, *a* = 6.35 Å (*a*_0_)), tetragonal (*I*4/*mcm*, *a* = √2*a*_0_; *c* = 2*a*_0_), and orthorhombic (**Pnma**, *a* = √2*a*_0_, *b* = 2*a*_0_, *c* = √2*a*_0_), and their Glazer notations are *a*^0^*a*^0^*a*^0^, *a*^0^*a*^0^*c*^–^, and *a*^+^*b*^–^*b*^–^, respectively.^48^ Whereas the tetragonal structure is generally favored at room temperature, the coexistence of different MAPbI_3_ structures has been found in nanocrystals and thin films.^49^

Selected-area ED (SAED) was used to examine the specimens with a very low electron dose rate (<0.1 e^–^/Å^2^s) to avoid beam-induced structural changes. In addition to the reflections associated with the primary phase of MAPbI_3_ at room temperature (i.e., tetragonal *I*4/*mcm*), super-reflections were locally observed in all three specimens oriented along the [001], [201], and [302] axes (Figure 5C–E), suggesting the presence of the secondary phase in the supposedly perfect single crystal of MAPbI_3_. To determine the unit cell parameters of the secondary phase, we collected 3D ED data from the area showing super-reflections by stepwise tilting the specimen within an angle range of ±45° (one ED pattern every 0.5°; 180 patterns total). The reconstructed reciprocal lattice exhibits a cubic unit cell with *a* = 12.75 Å (Figure S10), twice that of the parent cubic structure (*Pm*3̅*m*, *a* = 6.35 Å) of MAPbI_3_. Based on this unit cell, the three SAED patterns with super-reflections are indexed as the [001], [111], and [221] of the secondary phase (Figure 5C–E). According to previous studies,^47,50^ the PbI_3_ framework of the secondary phase should adopt the *a*^+^*a*^+^*a*^+^ tilt mode belonging to the *Im*3̅ space group. The observed reflections do not follow the body-centered lattice but the primitive lattice, which can be attributed to the presence of low-symmetry and disordered MA cations. Figure 5G illustrates the different PbI_3_ tilting modes in the primary tetragonal and secondary cubic phases and their geometrical relationships with the parent structure (*Pm*3*®m*, *a* = 6.35 Å).

The cryo-FIB-prepared specimen also allowed SAED to locate the two phases precisely, visualizing their distributions in a large area. In the [302]_T_/[221]_C_ direction, super-reflections appear in rows and are easy to identify (Figure 5E); thus, we chose to use the specimen of this orientation for SAED mapping, which was performed by acquiring a series of SAED patterns in a raster fashion over the entire specimen with 500 nm intervals. The results revealed that the secondary phase characterized by the “superlattice” was concentrated in the upper left corner of the specimen, spanning an area of about 2.5 × 2 μm (Figure 5F). Ultralow-dose HRTEM image taken from this region showed that the secondary phase formed irregular domains (10–20 nm) randomly distributed in the primary phase (Figure S11). This study again demonstrates the unique power of cryo-FIB in revealing local structures in sensitive bulk materials.

## Conclusions

In summary, cryo-FIB inherits the advantages of the traditional FIB while minimizing the damage effects of the ion beam, making it a powerful tool for preparing (S)TEM specimens for highly sensitive materials. With the assistance of cryo-FIB, the emerging ultralow-dose (S)TEM techniques are no longer limited to imaging nanosized samples but can be used to unravel local structures hidden in bulk crystals and devices comprising sensitive materials. The results of several case studies discussed in this paper demonstrate the effectiveness of cryo-FIB in different application scenarios and its compatibility with various electron microscopy modes. The combination of cryo-FIB and ultralow-dose (S)TEM revealed the structures of planar defects and the concomitant phase in large MOF crystals and the distribution of the superlattice in a hybrid perovskite single-crystal device. It is almost impossible for other characterization methods to obtain these structural details with such high spatial resolution. The current results demonstrate that ion beams with lower energy do not necessarily cause less damage to the structure. More in-depth research is needed to better understand the damage effects of ion beams on sensitive structures at cryogenic temperatures, which may vary with the nature of the material. In addition, caution should be exercised when applying cryo-FIB to temperature-sensitive materials that may undergo irreversible structural changes upon cooling at cryogenic temperatures.

## Supporting Information Available

Detailed operational processes are described as Experimental details, Figures S1–S11, and Table S1 (PDF)
Structural model for planar defects frequently observed in HKUST-1 (PDB)
Structural model for the interface between UiO-66 and Zr_12_-BDC (PDB)

## Supplementary Material

ja1c12794_si_001.pdf

ja1c12794_si_002.pdb

ja1c12794_si_003.pdb
