# Supplementary material for: Cryogenic Focused Ion Beam Enables Atomic-Resolution Imaging of Local Structures in Highly Sensitive Bulk Crystals and Devices
Source: J Am Chem Soc. 2022 Feb 14;144(7):3182–91. doi: 10.1021/jacs.1c12794 (PMC8874919; doi:10.1021/jacs.1c12794)
Supplement: Supplementary file 1 — ja1c12794_si_001.pdf [file ja1c12794_si_001.pdf]

Supporting Information

Cryogenic Focused Ion Beam Enables Atomic-resolution Imaging of Local Structures in Highly Sensitive Bulk Crystals and Devices

Jinfei Zhou[1, ‡], Nini Wei[1, ‡], Daliang Zhang[1,*], Yujiao Wang[1], Jingwei Li[1], Xiaopeng Zheng[2], Jianjian Wang[1], Abdullah Y. Alsalloum[2], Lingmei Liu[1], Osman M. Bakr[2], Yu Han[3,*]

[1] Multi-scale Porous Materials Center, Institute of Advanced Interdisciplinary Studies & School of Chemistry and Chemical Engineering, Chongqing University; Chongqing, 400044 P. R. China.

[2] King Abdullah University of Science and Technology (KAUST), Physical Sciences and Engineering Division; Thuwal, 23955-6900, Saudi Arabia.

[3] King Abdullah University of Science and Technology (KAUST), Physical Sciences and Engineering Division, Advanced Membranes and Porous Materials (AMPM) Center; Thuwal 23955-6900, Saudi Arabia.

‡These authors contributed equally to this work.

* Correspondence: daliang.zhang@cqu.edu.cn; yu.han@kaust.edu.sa

Materials

The micron-sized HKUST-1 crystals and millimeter-sized $CH_3NH_3PbI_3$ crystals were synthesized according to two previously reported methods.[1, 2] The core-shell structured Zr-BDC MOF crystals were synthesized using the following procedure. In a 10 mL vial, 24 mg (0.074 mmol) of zirconium oxychloride octahydrate was dissolved in 2 mL of N,N-diethylformamide (DEF). Separately, 10 mg (0.06 mmol) of terephthalic acid was dissolved in 2 mL of DEF. The two solutions were mixed, and then 4 mL of formic acid was added. The obtained milk-like suspension was sonicated for 1 h to obtain a clear solution, which was put into an autoclave and placed in an oven at 135°C for two days. The crystals were collected by centrifugation, washed with DMF and methanol, and then dried under a vacuum at 80 °C.

The single-crystal $CH_3NH_3PbI_3$ perovskite solar cell with a p-i-n planar configuration was structured as indium tin oxide glass substrate/poly(triarylamine) (PTAA)/perovskite/fullerene (C_{60})/bathocuproine (BCP)/Cu. The approximately 20 µm $CH_3NH_3PbI_3$ single-crystal thin films were grown on the PTAA hole transport layer using a previously reported space-limited method.[3, 4] Specifically, 60 µL of 1.6 M of $MAPbI_3$ in γ-Butyrolactone was dropped on a PTAA-coated substrate (5 × 5 cm) at 60°C and covered by another same-size PTAA-coated substrate. The temperature was gradually increased from 60°C to 120°C at a rate of 3°C/h. The substrates were then separated and slowly cooled to room temperature. The devices were finished by thermally evaporating BCP (15 nm), C_{60} (20 nm) and Cu (80 nm) in sequential order using a thin-film vacuum deposition system.

Cryo-FIB specimen preparation

The (S)TEM specimen preparations were performed on an FEI Helios 5 dual-beam microscope equipped with a cryogenic system (Cryo-mat). The cryo-FIB procedure for millimeter-sized crystals and devices is slightly different from that for micro-sized crystals, so we describe them separately.

1) Specimen preparation for micron-sized crystals (HKUST-1 and UiO-66@Zr_{12}-BDC)

The studied crystals were dispersed on a silicon wafer fixed on the cryo-stage. Electron beam-induced Pt deposition was employed to connect the selected crystal to the probe needle and later onto the TEM grid. During Pt deposition, the electron beam only irradiated the connection point and did not cause damage to the rest of the crystal. After the crystal was firmly attached to the TEM grid, the probe needle was cut off using the (Ga^+) ion beam. The orientation of the crystal relative to the TEM grid was controlled by combining the probe needle rotation, stage rotation, and stage tilt.

After the selected crystal was mounted on the TEM grid, the cryo-stage was gradually cooled to about -140°C by supplying liquid N_2. The stage slightly moved away from the eucentric position and the gas injection tube to ensure that the top crystal surface exposed to the ion beam could be covered by the organometallic Pt precursor. The cold surface of the crystal significantly promotes the adsorption and deposition of the precursor forming a thick organometallic layer (about 5 µm) within 2 min. The stage was then tilted by 52° to align the TEM grid with the incidence of the ion beam, which was subsequently used to cure the organometallic precursor to form a Pt-C protective layer.

After the protective layer was formed, the stage was finely tilted to perfectly align the desired direction of the crystal with respect to the incidence of the ion beam. A milling pattern was drawn around the region of interest, based on which the crystal was sectioned into a 3-µm-thick lamella using an ion beam (accelerating voltage: 30 kV, beam current: 2.5 nA) and further thinned to 1 µm with a lower beam current of 0.43 nA. Lastly, the lamella was finely milled to a thickness of less than 100 nm using an ion beam at 16 kV with a gentle beam current of 0.22 nA.

The stage was heated to 55°C at a rate of 5°C/min and kept for 30 min. Finally, the prepared specimen was collected from the FIB and transferred immediately to the TEM microscope for imaging.

2) Specimen preparation from bulk samples (millimeter-sized $CH_3NH_3PbI_3$ crystal and solar cell)

After the bulk sample (crystal or device) was placed on the cryo-stage, the cryo-stage was gradually cooled to about -140°C by supplying liquid N_2. The stage was slightly moved away from the eucentric height and gas injection tube to ensure that the organometallic Pt precursor could cover a large surface area. The stage was then tilted by 52° to align the specimen surface with the incidence of the (Ga^+) ion beam, which was subsequently used to cure the organometallic precursor to form a Pt-C protective layer. Then, a prism-shaped specimen (about 10 µm long, 5 µm wide, and 5 µm deep) was ablated using the ion beam (30 kV and 21 nA). The stage was tilted back to 0°, and a series of undercuts and side cuts (the U-cut) was then performed to free the "prism" specimen from the bulk sample.

The probe needle was carefully moved to touch one side of the "prism." A small region of the probe needle was milled with the ion beam to connect it with the "prism" through redeposition. Then, the "prism" specimen was cut off

from the bulk sample and lifted out by the probe needle. The specimen was transferred to a TEM grid by the probe needle and mounted on the grid by ion-beam milling of the Cu skeleton through the redeposition effect. The subsequent sectioning, fine milling, and specimen transfer processes were the same as those used for micron-sized crystals (described above).

Ultralow-dose HRTEM imaging and image processing

Ultralow-dose HRTEM was performed on a Cs-corrected electron microscope (FEI Titan) operated at 300 kV. The spherical aberration was corrected to a range of ± 5 μm. Specimen searching, zone axis alignment, and prefocusing were conducted at a 13,000× magnification with a dose rate of about 0.03 e/Å^2/s. The HRTEM images were collected using a Gatan K2 direct-detection camera in the electron-counting mode. The total electron dose used for each image was less than 15 e/Å^2 to avoid structural damage. The images were processed using CTF correction to be more directly interpretable. The detailed methods for image acquisition and processing (image alignment, determination of the absolute defocus value, and CTF correction) can be found in earlier publications.[5, 6]

Ultralow-dose iDPC-STEM imaging

Ultralow-dose iDPC-STEM was performed on a double Cs-corrected electron microscope (FEI Spectra 300) operated at 300 kV. Specimen searching and zone axis alignment were conducted at a 13,000× magnification under the TEM mode. Following the alignment of the crystal zone axis, the microscope was switched to the STEM mode with a convergence semi-angle of 10.0 mrad to acquire images. The probe current was about 1 pA, and the pixel size was 0.3716 Å. The dwell time was 5 μs/pixel, and the collection angle was 4-15 mrad. A four-quadrant DF4 detector was used to produce iDPC-STEM images and an applied high-pass filter to reduce low-frequency information.

Selected-Area Electron Diffraction (SAED) mapping

SAED patterns were recorded in a raster fashion over the cryo-FIB-prepared specimen (step size: 500 nm; 15×10 steps), using an aperture of about 400 nm, and a Gatan Ultra-scan CCD camera. Each pattern had a frame size of 512×512 (binning 4) and an exposure time of 2 s. The automatic acquisition of the SAED series was achieved through GMS scripting. During the SAED mapping process, the electron dose received by the specimen was less than 1 e/Å^2.

Three-Dimensional Electron Diffraction

The 3D ED data was collected with a step-wise rotation method, using a selected area aperture of ~400 nm, a constant stage rotation interval of 0.5°, an exposure time of 2 s for each pattern, and a Gatan Oneview camera. The rotation angle range was ±45°. Software REDp was used to reconstruct the reciprocal lattice and determine the extinction rules.

Powder X-ray diffraction

Powder X-ray diffraction patterns were recorded using a Bruker D8 Advance instrument with Cu Kα radiation (λ = 0.1542 nm) operated at 40 kV and 40 mA with a scanning speed of 0.5°/min.

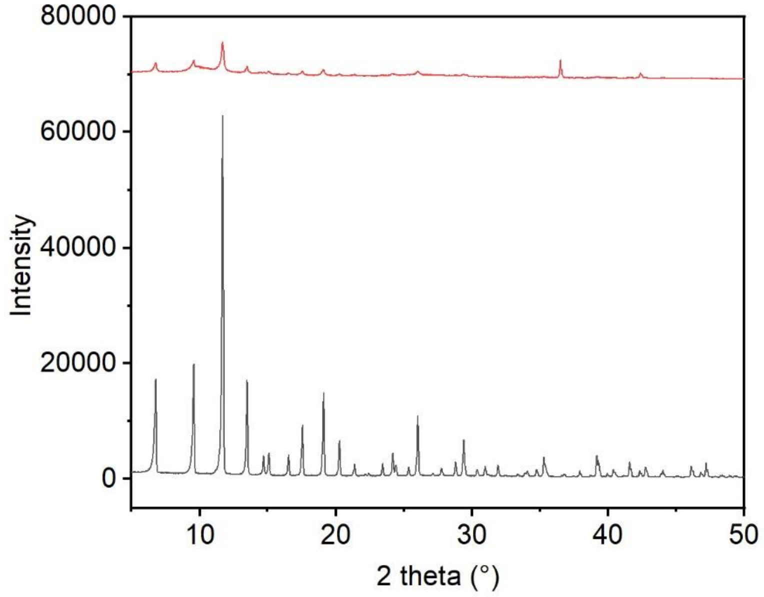

Figure S1.
Powder X-ray diffraction patterns of MOF HKUST-1 crystals before (black) and after grinding (red).

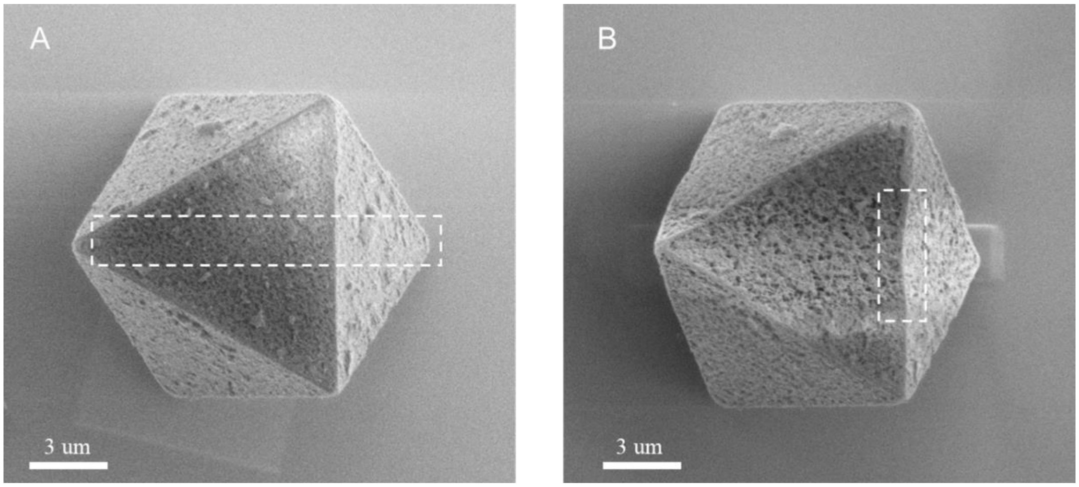

Figure S2.
SEM images of a MOF HKUST-1 crystal (**A**) before and (**B**) after Pt deposition at room temperature. The dashed rectangle in (**A**) indicates the region of Pt deposition. An electron beam (5 kV and 2.8 nA) was used to decompose the Pt precursor. Due to the low adsorption efficiency of the Pt precursor at room temperature, it takes time to form a Pt protection layer of sufficient thickness on the crystal surface. In (**B**), the region marked by the dashed rectangle displays obvious deformation after 4 min of Pt deposition, whereas the Pt layer has not yet been fully formed at this time.

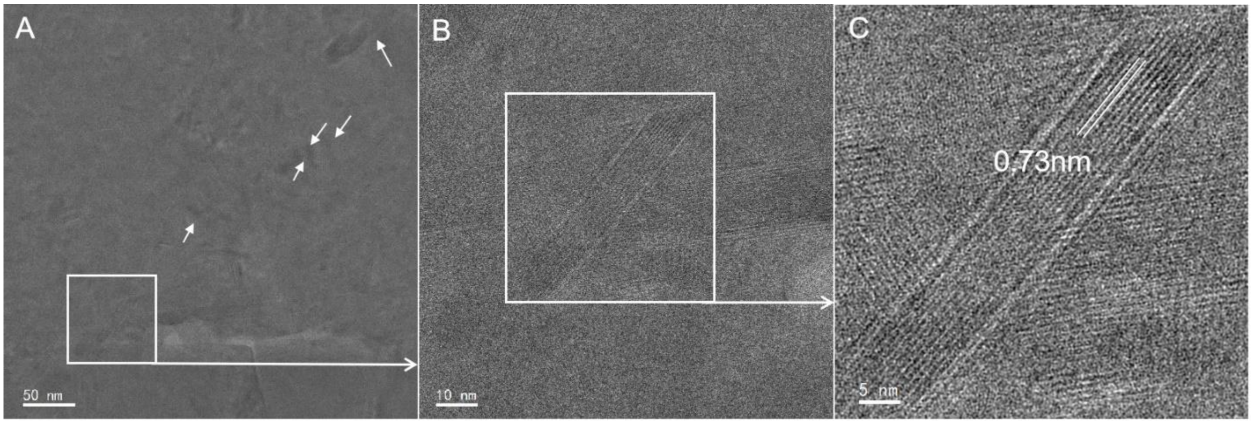

Figure S3.
Ultralow-dose HRTEM images of a specimen extracted from a $MAPbI_3$ single crystal using conventional FIB operated at room temperature. (**A**) Raw image acquired with an electron dose of 20 e/Å^2, (**B**-**C**) Cropped images at different magnifications, presenting the formation of PbI_2 caused by the ion beam-induced phase transition, where the lattice fringes with the *d* spacing of 0.73 nm correspond to the (003) crystal planes of PbI_2.[7] Arrows in (A) indicate other PbI_2 crystals produced by the ion-beam irradiation.

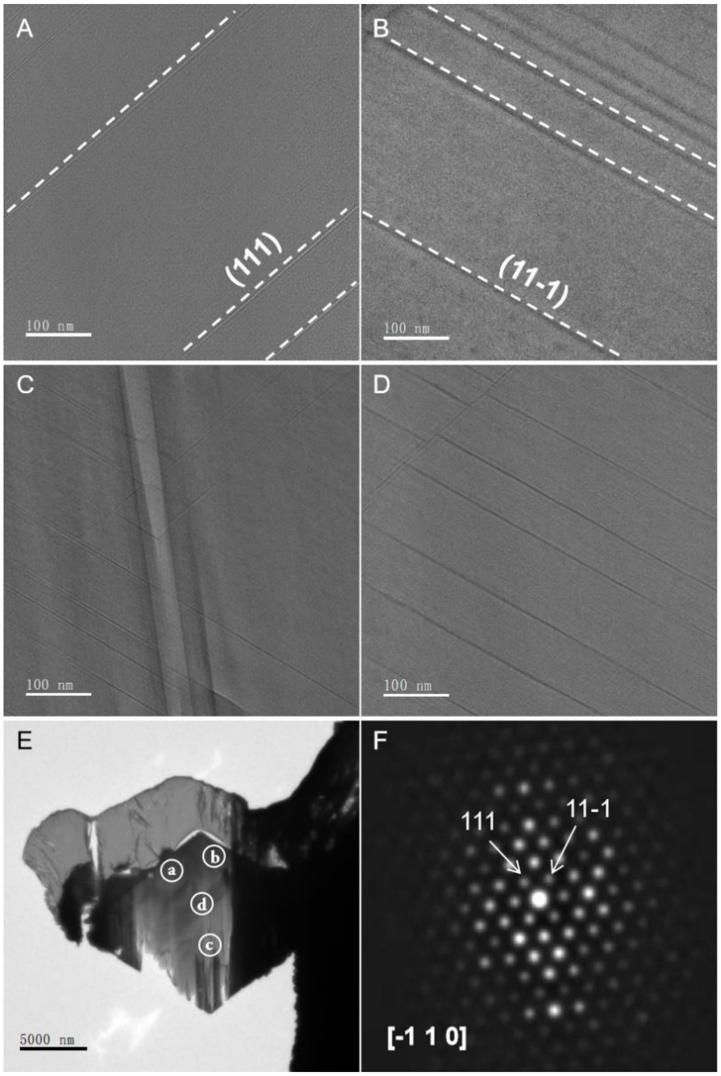

Figure S4.
(**A**-**D**) Ultralow-dose HRTEM images of HKUST-1 taken from different areas in the cryo-FIB-prepared <110>-oriented specimen, revealing abundant and ubiquitous {111} planar defects. (**E**) Low-magnification TEM image of the specimen, in which the labeled areas correspond to the HRTEM images with the same symbols. (**F**) SAED pattern from this specimen.

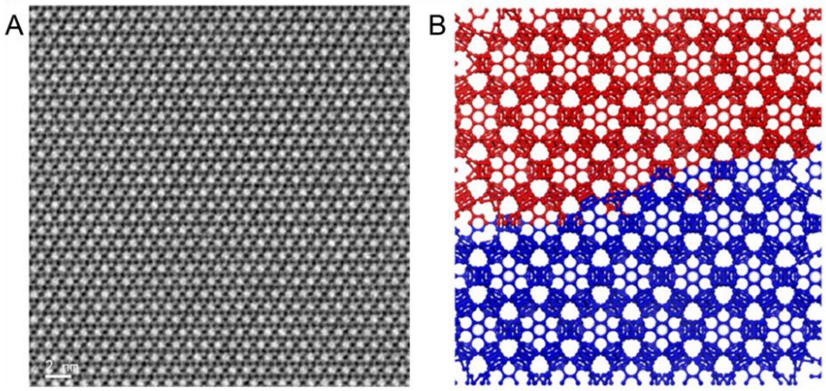

Figure S5.
(**A**) Ultralow-dose HRTEM image of HKUST-1 from the cryo-FIB-prepared <111>-oriented specimen. (**B**) The proposed structural model for the {111} planar defect in HKUST-1, projected along the <111> direction. The HRTEM image exhibits no structural defects in this projection direction, which agrees well with the proposed structural model.

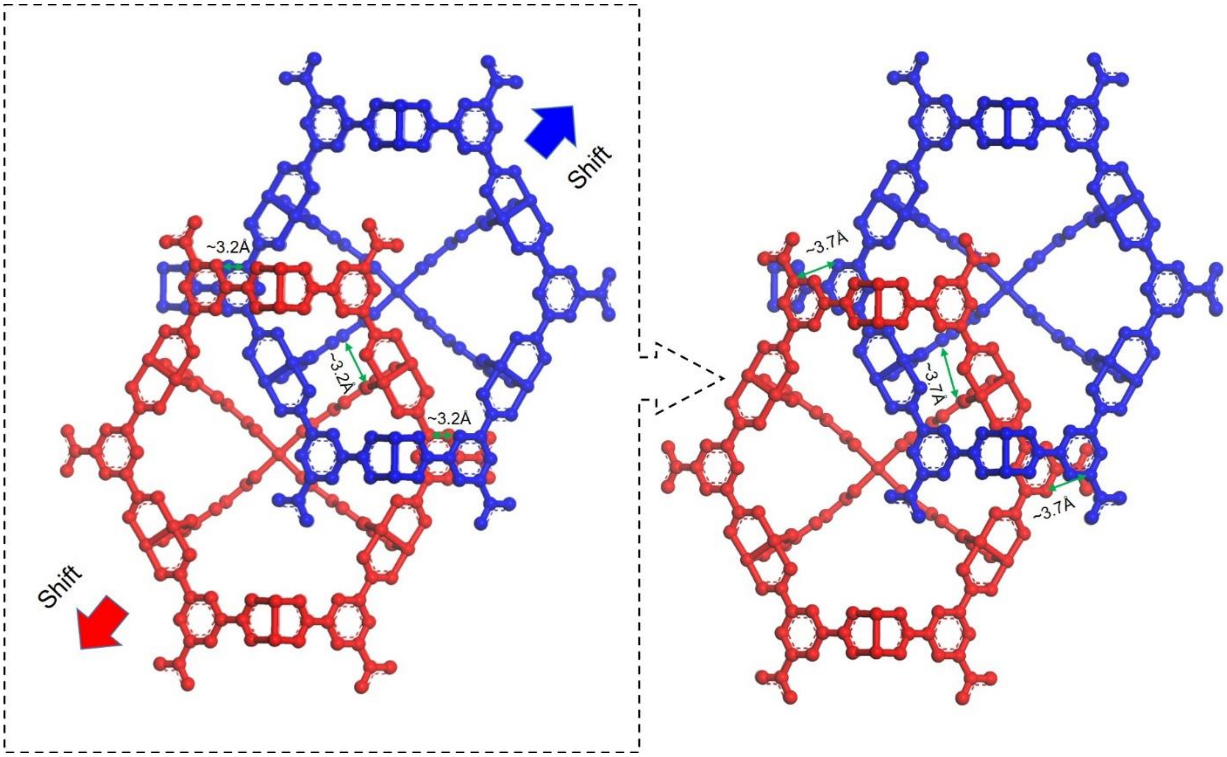

Figure S6.
Effect of the interpenetration depth of two HKUST-1 lattices on the distance between the aromatic rings of the BTC ligands at the interface.

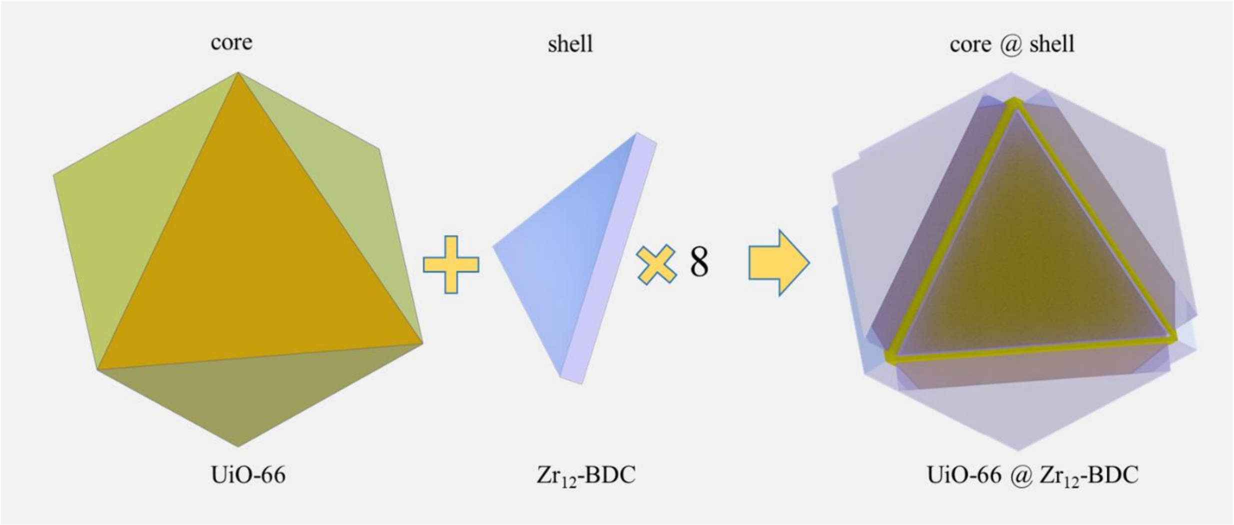

Figure S7.
Schematic illustration of the core-shell MOF structure formed by the intergrowth of cubic UiO-66 (the octahedral core) and hexagonal Zr_{12}-BDC (shell grown on eight core surfaces).

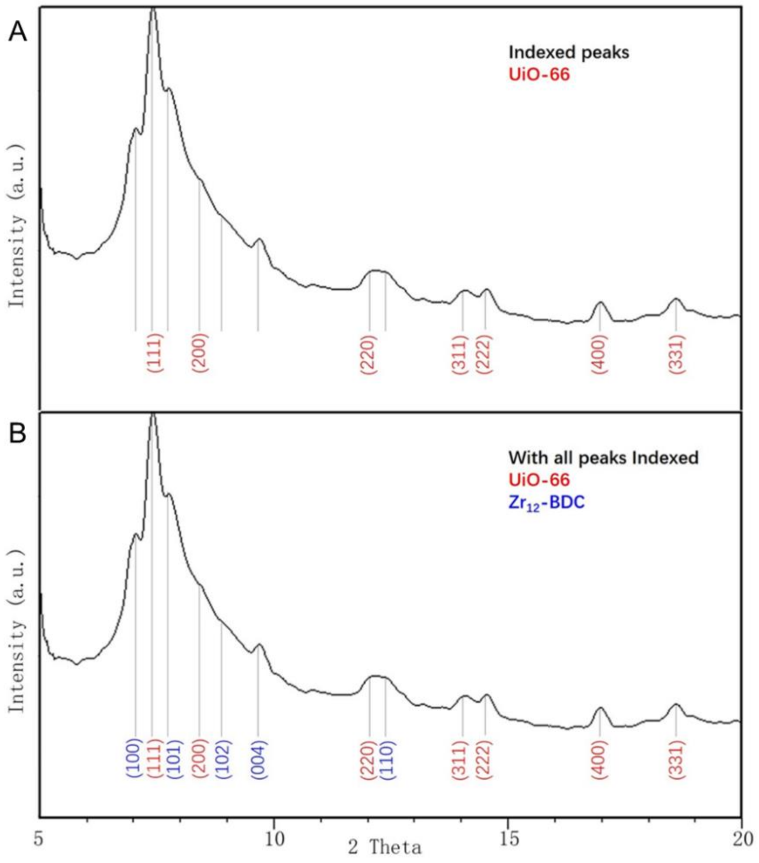

Figure S8.
Powder X-ray diffraction pattern of the UiO-66 core-shell structure. (**A**) Powder X-ray diffraction pattern of the core-shell structured MOF, revealing the presence of unidentifiable peaks in addition to those associated with UiO-66. (**B**) The same pattern with all peaks indexed. Indexing is achieved after the shell structure is determined by combining cryo-FIB and ultralow-dose iDPC-STEM.

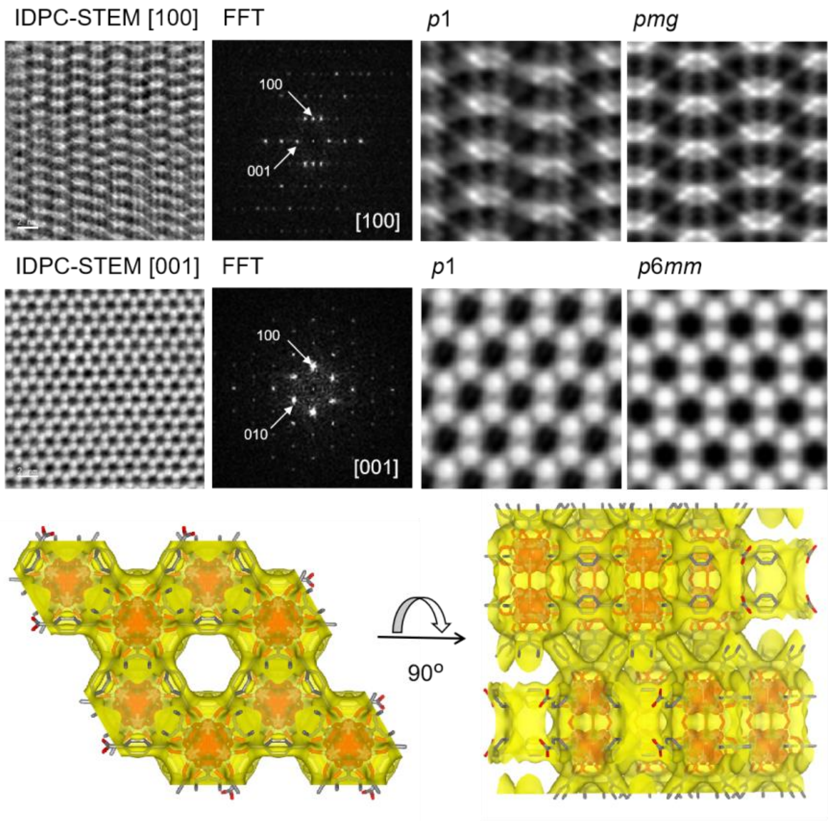

Figure S9.
Electron crystallography reconstruction of Zr_{12}-BDC based on the iDPC-STEM images. The iDPC-STEM images along the [100] and [001] zone axes were processed using Crisp software. The projection symmetry of *pmg* and *p*6*mm* were determined for the [100] and [001] zone axes, respectively, with φ_{res} < 10%. The amplitudes and phases of structure factors extracted from reflections were merged to produce the 3D electrostatic potential map through Fourier synthesis.

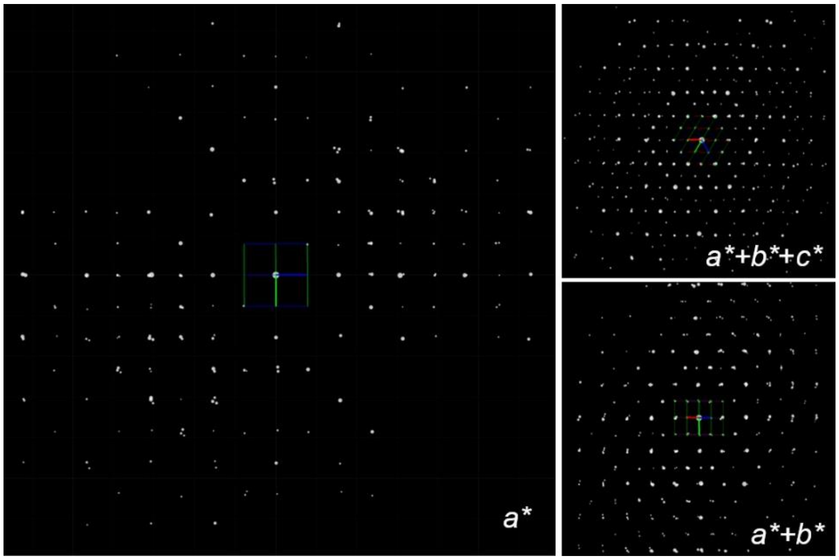

Figure S10.
Reciprocal lattice of single-crystal $MAPbI_3$ in a solar cell, viewed in different directions. The reciprocal lattice was constructed using 3D electron diffraction, from which a cubic unit cell with *a* = 12.75 Å was determined. The lattice does not show systematic absence.

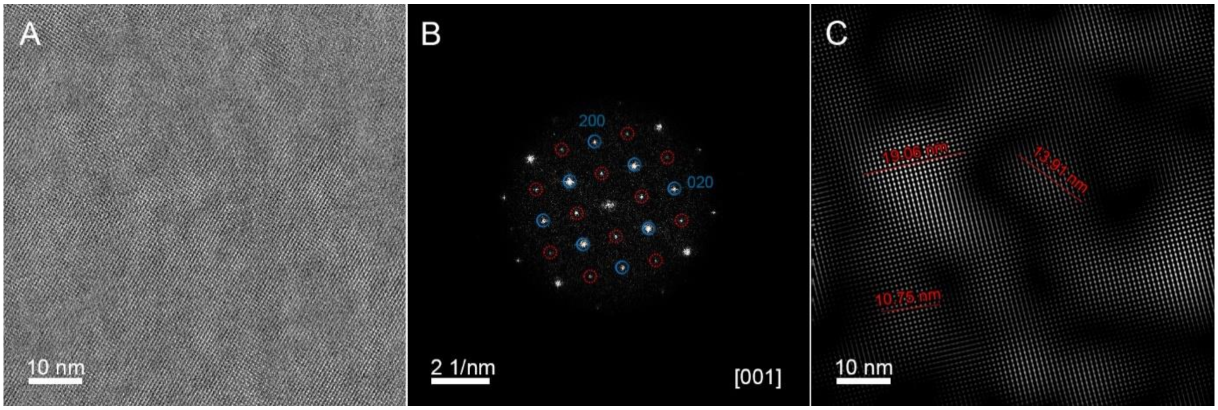

Figure S11.
(**A**) HRTEM image taken from a region showing super reflections. (**B**) Fourier transform of the image with reflections of the primary and secondary phases marked by blue and red circles, respectively. (**C**) Fourier filtered image using super reflections with d values greater than 0.8 nm, showing the sizes and distribution of the secondary phase domains.

Table S1.
Experimental structure factors of Zr_{12}-BDC extracted from iDPC-STEM images

h	*k*	*l*	*Amp.*	*Phase*
0	1	1	10,000	0
0	1	0	7,338	180
0	0	2	6,303	180
0	0	4	3,519	180
0	2	4	2,720	0
0	0	6	2,072	0
0	1	3	1,898	0
0	1	2	1,483	0
0	2	1	1,153	180
0	3	3	951	180
0	3	7	929	0
0	2	5	907	0
0	1	4	745	0
0	3	5	678	0
0	2	8	663	180
0	3	6	585	0
0	3	9	544	180
0	2	2	539	0
0	1	5	498	180
0	2	6	497	0
0	3	0	467	180
0	1	6	408	180
0	4	4	396	180
0	1	7	385	180
0	2	3	362	0
0	2	7	346	180
0	4	2	344	0
0	1	10	341	180
0	1	11	329	0

References

1. Wang, F. L.; Guo, H. L.; Chai, Y. M.; Li, Y. P.; Liu, C. G. The controlled regulation of morphology and size of HKUST-1 by "coordination modulation method". *Microporous Mesoporous Mater.* **2013,** *173*, 181–188. DOI:10.1016/j.micromeso.2013.02.023
2. Saidaminov, M. I.; Abdelhady, A. L.; Murali, B.; Alarousu, E.; Burlakov, V. M.; Peng, W.; Dursun, I.; Wang, L. F.; He, Y.; Maculan, G.; Goriely, A.; Wu, T.; Mohammed, O. F.; Bakr, O. M., High-quality bulk hybrid perovskite single crystals within minutes by inverse temperature crystallization. *Nature Communications* **2015,** *6*, 7586 DOI: 10.1038/ncomms8586
3. Chen, Z. L.; Dong, Q. F.; Liu, Y.; Bao, C. X.; Fang, Y. J.; Lin, Y.; Tang, S.; Wang, Q.; Xiao, X.; Bai, Y.; Deng, Y. H.; Huang, J. S., Thin single crystal perovskite solar cells to harvest below-bandgap light absorption. *Nature Communications* **2017,** *8*, 1890. DOI: 10.1038/s41467-017-02039-5
4. Alsalloum, A. Y.; Turedi, B.; Almasabi, K.; Zheng, X. P.; Naphade, R.; Stranks, S. D.; Mohammed, O. F.; Bakr, O. M., 22.8%-Efficient single-crystal mixed-cation inverted perovskite solar cells with a near-optimal bandgap. *Energy & Environmental Science* **2021,** *14* (4), 2263-2268. DOI: 10.1039/d0ee03839c
5. Zhang, D. L.; Zhu, Y. H.; Liu, L. M.; Ying, X. R.; Hsiung, C. E.; Sougrat, R.; Li, K.; Han, Y., Atomic-resolution transmission electron microscopy of electron beam-sensitive crystalline materials. *Science* **2018,** *359* (6376), 675-679. DOI: 10.1126/science.aao0865
6. Liu, L. M.; Chen, Z. J.; Wang, J. J.; Zhang, D. L.; Zhu, Y. H.; Ling, S. L.; Huang, K. W.; Belmabkhout, Y.; Adil, K.; Zhang, Y. X.; Slater, B.; Eddaoudi, M.; Han, Y., Imaging defects and their evolution in a metal-organic framework at sub-unit-cell resolution. *Nature Chemistry* **2019,** *11* (7), 622-628. DOI: 10.1038/s41557-019-0263-4
7. Chen, S. L.; Zhang, X. W.; Zhao, J. J.; Zhang, Y.; Kong, G. L.; Li, Q.; Li, N.; Yu, Y.; Xu, N. G.; Zhang, J. M.; Liu, K. H.; Zhao, Q.; Cao, J.; Feng, J. C.; Li, X. Z.; Qi, J. L.; Yu, D. P.; Li, J. Y.; Gao, P., Atomic scale insights into structure instability and decomposition pathway of methylammonium lead iodide perovskite. Nature Communications 2018, 9, 4807. DOI: 10.1038/s41467-018-07177-y
